# Oscillatory Disturbed Flow Enhances Inflammatory and Oxidative Stress Markers in Endothelial Cells

**DOI:** 10.3390/mps8060130

**Published:** 2025-11-01

**Authors:** Maram Hasan, Onur Mutlu, Munshi Sajidul Islam, Samar Shurbaji, Ruba Sulaiman, Yasmin Elsharabassi, Abdelali Agouni, Huseyin C. Yalcin

**Affiliations:** 1 Biomedical Research Center, Qatar University, QU Health, Doha P.O. Box 2713, Qatar; mhasan@qu.edu.qa (M.H.); onurmutlu94@gmail.com (O.M.); sajidul.islam@qu.edu.qa (M.S.I.); ss1104227@student.qu.edu.qa (S.S.); rs1607806@student.qu.edu.qa (R.S.); 2 College of Dental Medicine, QU Health, Qatar University, Doha P.O. Box 2713, Qatar; 3 Collage of Health Sciences, Qatar University, Doha P.O. Box 2713, Qatar; ye1701976@student.qu.edu.qa; 4 Department of Pharmaceutical Sciences, College of Pharmacy, QU Health, Qatar University, Doha P.O. Box 2713, Qatar; aagouni@qu.edu.qa; 5 Department of Biomedical Sciences, College of Health Sciences, QU Health, Qatar University, Doha P.O. Box 2713, Qatar; 6 Department of Mechanical and Industrial Engineering, Qatar University, Doha P.O. Box 2713, Qatar

**Keywords:** atherosclerosis, disturbed flow, endothelial dysfunction, shear stress

## Abstract

Hemodynamics significantly impact the biology of endothelial cells (ECs) lining the blood vessels. ECs are exposed to various hemodynamic forces, particularly frictional shear stress from flowing blood. While physiological flows are critical for the normal functioning of ECs, abnormal flow dynamics, known as disturbed flows, may trigger endothelial dysfunction leading to atherosclerosis and other vascular conditions. Such flows can occur due to sudden geometrical variations and vascular abnormalities in the cardiovascular system. In the current study, a microfluidic system was used to investigate the impact of different flow conditions (i.e, normal vs. disturbed) on ECs in vitro. We particularly explored the relationship between specific flow patterns and cellular pathways linked to oxidative stress and inflammation related to atherosclerosis. Here, we utilized a 2D cell culture perfusion system featuring an immortalized human vascular endothelial cell line (EA.hy926) connected to a modified peristaltic pump system to generate either steady laminar flows, representing healthy conditions, or disturbed oscillatory flows, representing diseased conditions. EA.hy926 were exposed to an oscillatory flow shear stress of 0.5 dynes/cm^2^ or a laminar flow shear stress of 2 dynes/cm^2^ up to 24 h. Following flow exposure, cells were harvested from the perfusion chamber for quantitative PCR analysis of gene expression. Reactive oxygen species (ROS) generation under various shear stress conditions was also measured using DCFDA/H2DCFDA fluorescent assays. Under oscillatory shear stress flow conditions (0.5 dynes/cm^2^), EA.hy926 ECs showed a 3.5-fold increase in the transcription factor nuclear factor (*NFκ-B*) and a remarkable 28.6-fold increase in cyclooxygenase-2 (*COX-2*) mRNA expression, which are both proinflammatory markers, compared to static culture. Transforming growth factor-beta (*TGFβ*) mRNA expression was downregulated in oscillatory and laminar flow conditions compared to the static culture. Apoptosis marker transcription factor Jun (*C-Jun*) mRNA expression increased in both flow conditions. Apoptosis marker C/EBP homologous protein (*CHOP*) mRNA levels increased significantly in oscillatory flow, with no difference in laminar flow. Endothelial nitric oxide synthase (*eNOS*) mRNA expression was significantly decreased in cells exposed to oscillatory flow, whereas there was no change in laminar flow. Endothelin-1 (*ET-1*) mRNA expression levels dropped significantly by 0.5- and 0.8-fold in cells exposed to oscillatory and laminar flow, respectively. ECs subjected to oscillatory flow exhibited a significant increase in ROS at both 4 and 24 h compared to the control and laminar flow. Laminar flow-treated cells exhibited a ROS generation pattern similar to that of static culture, but at a significantly lower level. Overall, by exposing ECs to disturbed and normal flows with varying shear stresses, significant changes in gene expression related to inflammation, endothelial function, and oxidative stress were observed. In this study, we present a practical, optimized system as an in vitro model that can be employed to investigate flow-associated diseases, such as atherosclerosis and aortic aneurysm, thereby supporting the understanding of the underlying molecular mechanisms.

## 1. Introduction

Endothelial cells (ECs) line the interior of the blood vasculature. ECs are closely influenced by hemodynamics via autocrine and paracrine signaling [1]. Continuous exposure of ECs to different hemodynamic forces in vivo, predominantly frictional shear stress, effectively controls almost all aspects of EC activity. It has been demonstrated both in vivo and in vitro that blood flow modulates the transcriptomic and epigenetic environments of ECs, as well as their functions, including proliferation, inflammation, and differentiation [2]. The cardiovascular system is a complex architecture of branched vessels with varying sizes and structures. As a result, different blood flows occur at various locations.

Additionally, irregularities within the system, due to congenital or later acquired defects, influence hemodynamics. While straight vessel regions are exposed to unidirectional high shear stress flows, known as laminar flows, curved and branched regions are exposed to disturbed flows, typically characterized by flow oscillations with low shear stress. It was observed that vascular diseases, such as atherosclerosis, predominantly occur in regions that were exposed to disturbed flows; hence, these regions are termed atheroprone regions [1]. Similarly, our group and others have shown that in aortic aneurysms, degeneration and eventual rupture of the aorta are closely linked with hemodynamics, as rupture usually happens in low wall shear stress and high oscillation regions [1,3,4]. These clinical observations suggest that disturbed hemodynamics influence endothelial dysfunction, which is the initial stage of atherosclerosis and aneurysm [3].

The mechanobiology of endothelial dysfunction has been investigated using in vivo models, such as rodent models and zebrafish [5]. In vitro models are also very useful, as they are efficient, easy to implement, and provide complete control over the experimental conditions. For such investigations, researchers should closely match the physiological conditions of endothelial cells; hence, dynamic culture under flow conditions is necessary. With the advancement of microfluidics technologies, several previous works have been conducted on endothelial cells cultured in perfusion chambers connected to flow pumps to induce different shear stress conditions [6]. These studies focused on the mechanobiology of healthy and disturbed flows on endothelial cell function. They used different flow dynamic systems such as cone-and-plate chambers, parallel plates, and diaphragm pumps, in addition to other systems [7,8,9]. Peristaltic pumps are also commonly used; however, the flow they generate is complex and therefore does not replicate physiological flows well [10]. In most of these studies, either a single pattern of flow and shear stress was investigated, or a single pattern of flow with varying magnitudes of shear stress was studied, while the exposure time ranged from 2 to 48 h. Moreover, most studies have focused on reactive oxygen species (ROS) generation and examined genes limited to a specific pathway [11]. Healthy laminar flows are characterized by high-shear stress, whereas low-shear stress and oscillations in these investigations are considered as disturbed flows.

In the current study, we designed a microfluidic system to expose cultured endothelial cells to various physiological and pathological flow conditions. This system is composed of a multichannel cell culture chamber and a modified peristaltic pump, which can generate either steady flows or oscillatory flows over ECs. We compared gene expression profiles of selected pathways that have not been extensively investigated, including ROS, endoplasmic reticulum (ER) stress response, antioxidants, inflammatory response, cell survival and apoptosis, and nitric oxide (NO) generation-related markers. Here, we present a successfully optimized model for investigating the mechanobiology of endothelial dysfunction. Furthermore, we linked the effect of specified flow patterns to antioxidant, anti-inflammatory, and ER stress pathways in ECs as measured by RT-PCR and ROS generation assays.

## 2. Materials and Methods

### 2.1. Cell Culture and Maintenance

The EA.hy926 endothelial cell line (ATCC.^®^. CRL-2922TM) was obtained from the American Type Culture Collection (ATCC, Manassas, VA, USA). Cells were kept in Dulbecco’s Modified Eagle’s Medium (DMEM) containing low glucose (1 g/L) and complemented with fetal bovine serum (FBS, 10%), L-glutamine (2 mM), penicillin (10,000 units), and streptomycin (10 mg/L). All reagents were obtained from Gibco (ThermoScientific, Waltham, MA, USA). Cells were incubated at 37 ◦C in a 95% humidified atmosphere saturated with 5% CO_2_. EA.hy926 cells (5 × 10^5^) were seeded on Microfluidic Rhombic Chamber Chip eP1fluidic design 221 (Microfluidic chipshop, Jena, Germany), to perform flow exposure experiments. Seeded chips were placed in the cell incubator at 37 °C with 5% CO_2_. After 24 h of incubation, cells in the microfluidic chip reached approximately 80% confluency. Then the cells were exposed to different flow patterns at a specific shear stress.

### 2.2. Flow Experiment Setup and Generation of Laminar and Oscillatory Flows

**Figure 1:**
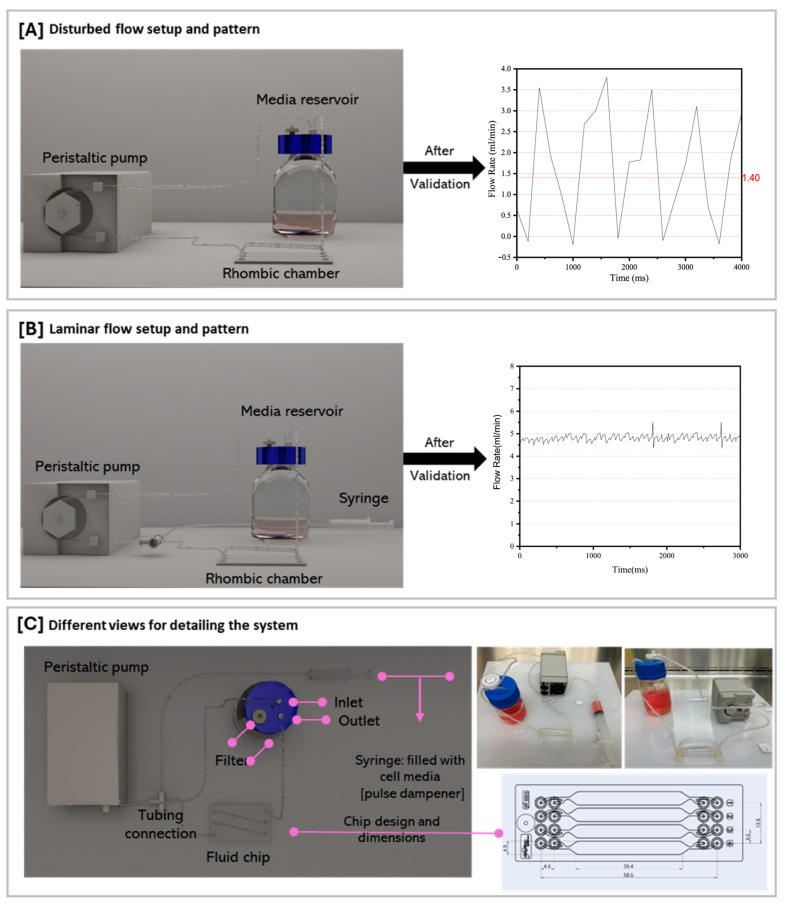
Peristaltic pump setup and associated flow rate measurement applied for inducing different flow patterns on cells cultured on a Microfluidic Rhombic Chamber Chip. (**A**) Disturbed flow setup and pattern, (**B**) laminar flow setup and pattern, and (**C**) different views for detailing the system.

ECs cultured within the microfluidic 2D cell culture system were subjected to different shear stress magnitudes and flow patterns. ECs were exposed to either oscillatory or steady laminar flow conditions. For oscillatory flow experiments, we utilized a peristaltic pump, specifically the Bioptechs Single Tube Micro-Perfusion Peristaltic Pump (Figure 1A). The oscillatory flow used in these experiments exhibits a periodic pattern with a constant frequency of ~1.1 Hz. As shown in Figure 1A, the flow rate alternates between increasing and decreasing values in a regular, repeating manner for the roller mechanism of the peristaltic pump. The peak amplitude reaches approximately ~3.8 mL/min in the positive direction, generating a 1.4 mL/min mean flow. The waveform resembles a triangular shape, with relatively linear increase and decrease in flow rate during each cycle. The single-tube pump inlet was connected to the media reservoir using a 1/16” Tygon tube, while the outlet was connected to the first inlet of the chip. In addition, another Tygon tube was placed in the media reservoir and connected to the chip’s last outlet. Small Tygon tubes were attached to the remaining openings in the chip, creating a closed circulation for the media to pass through the chip and back to the media reservoir. The peristaltic pump is battery-operated, and the entire system was kept on a tray inside the cell culture experiment throughout the flow experiments. A 0.2 µm filter was placed in one of the bottle cap openings to provide CO_2_ and air to the system and to prevent cell contamination. To generate a steady laminar flow (without oscillations), a 10 mL fluid-filled (media) syringe was connected to the Bioptechs FCS2 Single Tube Pump for dampening the pulses (Figure 1B).

To conduct the experiments involving an oscillatory flow pattern, the pump was functioning at a low speed to generate a fluctuating flow profile (Figure 1A). Regarding steady laminar flow pattern experiments, the pump was functioning at a higher speed to generate a constant-speed flow profile (Figure 1B). Hence, for oscillatory flow experiments, the mean flow rate in the microfluidic channels is about 1.4 mL/min, while for steady flow experiments, it is about 4.8 mL/min. The wall shear stress that the cells are exposed to within the microfluidic channel can be calculated from the following Poiseuille flow formula [12]:

$$\tau =\frac{6\eta Q}{h^{2}\omega }$$

Here, *Q* represents the volumetric flow rate, η is the dynamic viscosity of the fluid, *h* is the distance between the plates (analogous to channel height), and ω is the width of the plates (corresponding to the channel width). The rhombic chamber used in this experiment is the Fluidic221 model; the chambers are 58.5 mm in length, with a 35.4 mm rectangular portion. The width (ω) of the chamber is 4.5 mm, and it has a depth (h) of 600 μm. These calculations are based on the viscosity of water at a temperature of 37 °C, which is 0.69 mPa·s. 1.4 mL/min flow generated 0.5 dynes/cm^2^ shear stress on ECs, whereas 4.8 mL/min flow generated 2 dynes/cm^2^ shear stress on ECs.

### 2.3. RNA Isolation and Gene Expression Analysis

At the experimental endpoint, cells were trypsinized and collected from the chamber. We then conducted total RNA extraction and cDNA synthesis for gene expression studies by quantitative PCR. We followed the manufacturer’s instructions to isolate RNA and performed cDNA synthesis using the PureLink™ RNA Mini Kit (ThermoScientific) and the SuperScript™ VILO™ cDNA Synthesis Kit (ThermoScientific), respectively. Target genes underwent cycles of amplification using an Applied Biosystems QuantStudio™ 6 Flex Real-Time PCR System (ThermoScientific) with SYBR^®^ Green PCR Master Mix (ThermoScientific). Analysis of data was performed using the ΔΔC_t_ comparative method. Relative mRNA expression of target genes was normalized to housekeeping gene expression (GAPDH) and presented as a fold-change. Human primer pairs were obtained from PrimerBank and manufactured by Integrated DNA Technologies (IDT, Coralville, IA, USA). The primer pair sequences used in this study are summarized in Table 1.

### 2.4. Measurement of ROS Production

In this experiment, cells were seeded on a sterile glass coverslip coated with collagen rat tail I (ThermoScientific, Waltham, MA, USA) and exposed to the designated shear stress and flow patterns using a Bioptechs FCS2 Flow Chamber, which provides a suitable area for fluorescent imaging. Steady 2 dynes/cm^2^ and oscillatory 0.5 dynes/cm^2^ flow conditions were generated for these experiments as well. The DCFDA/H2DCFDA Cellular ROS Assay Kit (Abcam, Cambridge, UK) was used to measure ROS generation. After 24 h of exposing the cells to specific flow conditions, cells were washed twice with 1X washing buffer provided in the kit, then cells were stained using diluted DCFDA solution for 45 min at 37 °C protected from light, cells then were washed and imaged using the EVOS M5000 using a filter set compatible with Fluorescein (Ex/Em = 490/525 nm). Tert-butyl Hydrogen Peroxide (tbHP) (50 µM) served as the assay positive control. Fluorescence intensity was quantified and analyzed using ImageJ 1.x software, where the corrected total cell formula was employed to account for the field background.

CTCF = IntegratedDensity − (Area × Mean of Background), cell fluorescence was normalized per cell area.

### 2.5. Statistical Analysis

Data are represented as mean ± standard error of the mean (S.E.M.), and *n* is the number of biological repeats. Statistical analyses were performed with GraphPad Prism^®^ 10.1.0 software, which employed one-way ANOVA followed by Tukey’s multiple comparison post hoc test. *p* ≤ 0.05 was considered statistically significant.

## 3. Results

### 3.1. Proinflammatory Markers Gene Expression

**Figure 2:**
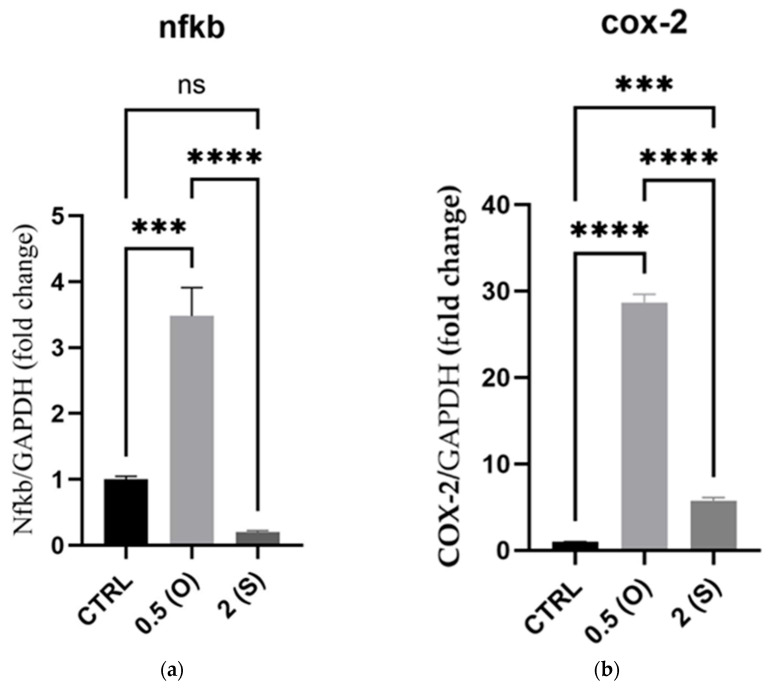
Effect of different flow patterns and shear stresses on mRNA expression of selected proinflammatory markers. Relative mRNA expression levels (fold change) of proinflammatory markers, *NFκ-B* (**a**), and *COX-2* (**b**), normalized against the *GAPDH* housekeeping gene (*n* = 4 in each group). Cells were exposed to two different flow conditions for 24 h. Data are presented as mean ± S.E.M. *** *p* < 0.001, **** *p* < 0.0001 versus CTRL or versus indicated groups. Treatment groups include CTRL, control: static culture with no flow; 0.5(O): oscillatory flow with low shear stress (0.5 dynes/cm^2^); 2(S): steady laminar flow with high shear stress (2 dynes/cm^2^).

Development of aortic aneurysms and atherosclerosis is facilitated by disturbed oscillatory blood flows, triggering ECs to express inflammatory genes and ROS generation, which in turn stimulates the expression and activity of proinflammatory markers, including the transcription factor nuclear factor (NF)κ-B and cyclooxygenase (COX)-2 [13,14]. As illustrated in Figure 2, *NFκ-B* and *COX-2* mRNA expression was significantly increased by 3.5- and 28.6-fold, respectively, under low oscillatory shear stress (0.5 dynes/cm^2^) flow conditions when compared to normal static culture. Under high steady flow (2 dynes/cm^2^), mRNA expression of *NFκ-B* was not affected; however, the *COX-2* mRNA level was significantly increased, albeit to a lesser extent, compared to the oscillatory flow conditions (Figure 2).

### 3.2. Transforming Growth Factor-Beta (TGFβ) Gene Expression

**Figure 3:**
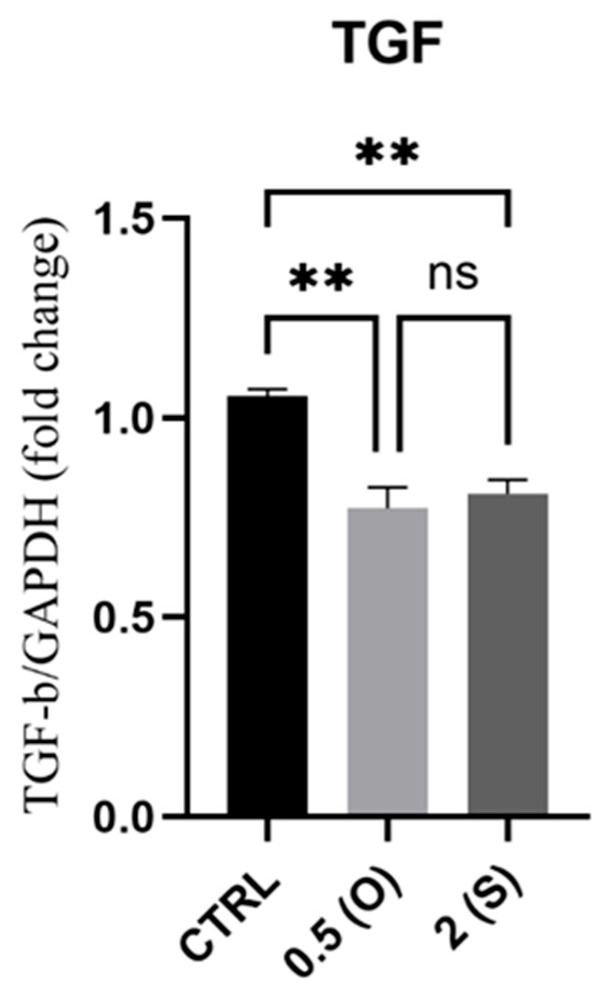
Effect of different flow patterns and shear stresses on mRNA expression of *TGFβ*. Relative mRNA expression levels (fold change) of *TGFβ*, normalized against *GAPDH* housekeeping gene (*n* = 3 in each group). Cells were exposed to two different flow conditions for 24 h. Data are presented as mean ± S.E.M. ** *p* < 0.01 versus CTRL. Treatment groups include CTRL, control: static culture with no flow; 0.5(O): oscillatory flow with low shear stress (0.5 dynes/cm^2^); 2(S): laminar flow with high shear stress (2 dynes/cm^2^).

Although profibrotic collagen deposition into the adventitia of the arterial wall during aging is linked to arterial stiffening, *TGF-β* appears to play a complex role in this process through TGF-β upregulation. The mRNA expression of *TGF-β* was measured under both oscillatory and steady flow conditions. As illustrated in Figure 3, *TGF-β* mRNA expression was downregulated under both flow conditions when compared to the control static culture.

### 3.3. Apoptosis and Cell Death Markers Gene Expression

**Figure 4:**
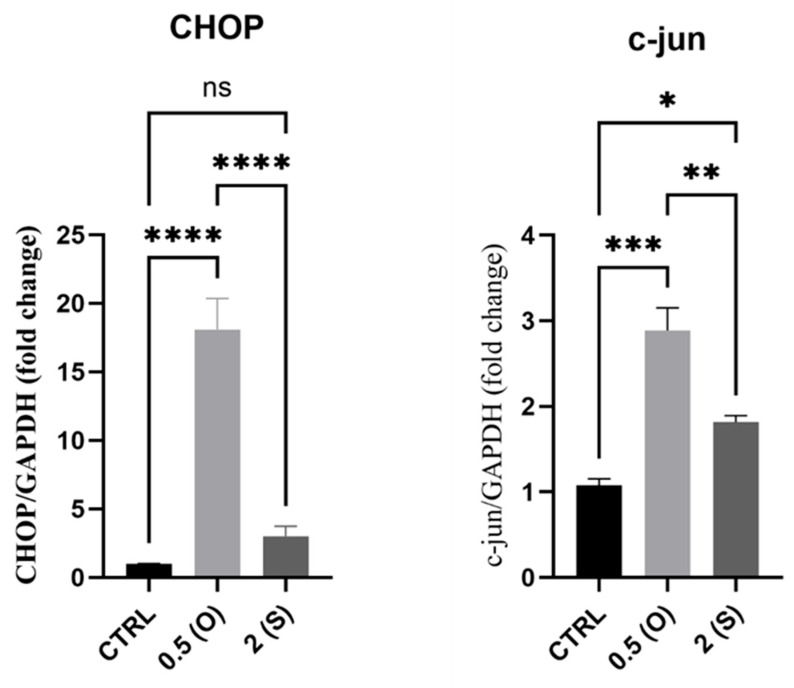
Effects of different flow patterns and shear stresses on mRNA expression of *CHOP* and *c-Jun*. Relative mRNA expression levels (fold change) of *CHOP* and *c-Jun*, normalized against the *GAPDH* housekeeping gene (*n* = 3 in each group). Cells were exposed to two different flow conditions for 24 h. Data are presented as mean ± S.E.M. * *p* < 0.05, ** *p* < 0.01, *** *p* < 0.001, **** *p* < 0.0001 versus CTRL or versus indicated groups. Treatment groups include CTRL, control: static culture with no flow; 0.5(O): oscillatory flow with low shear stress (0.5 dynes/cm^2^); 2(S): laminar flow with high shear stress (2 dynes/cm^2^).

It is known that, when subjected to disturbed flows, the ER of ECs fails to fold proteins correctly; instead, stress receptor-bound proteins are released to initiate an apoptotic signaling response termed ER stress, which may lead to reduced cell survival through the activation of inflammatory and cell death signals [1]. One of the major signaling molecules involved in ER stress-mediated cell death is CHOP [15]. As shown in Figure 4, *CHOP* mRNA levels increased significantly in endothelial cells exposed to low-shear stress and disturbed flow. At the same time, there was no difference in mRNA expression in cells exposed to laminar flow. C-Jun (one of the activation protein 1 (AP-1) family of transcription factors) mRNA levels also increased in both different flow patterns.

### 3.4. eNOS and ET-1 Gene Expression

**Figure 5:**
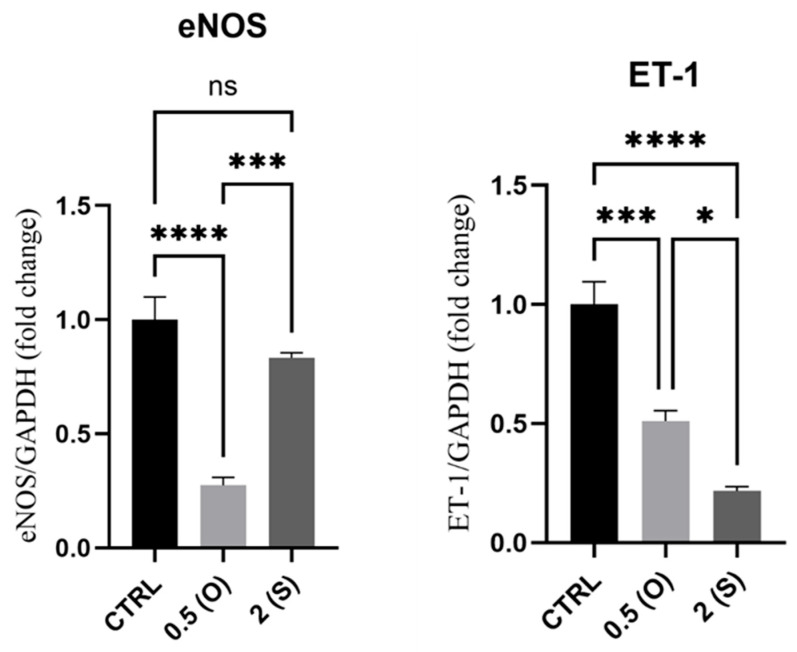
Effects of different flow patterns and shear stresses on mRNA expression of *eNOS* and *ET-1*. Relative mRNA expression levels (fold change) of *eNOS* and *ET-1*, normalized against the *GAPDH* housekeeping gene (*n* = 4 in each group). Cells were exposed to two different flow conditions for 24 h. Data are presented as mean ± S.E.M. * *p* < 0.05, *** *p* < 0.001, **** *p* < 0.0001 versus CTRL or versus indicated groups. Treatment groups include CTRL, control: static culture with no flow; 0.5(O): oscillatory flow with low shear stress (0.5 dynes/cm^2^); 2(S): laminar flow with high shear stress (2 dynes/cm^2^).

The endothelial NO synthase (eNOS) enzyme is essential for the induction of NO production, while ET-1 is an endogenous vasoconstrictor that, together with eNOS, preserves the vascular tone of blood vessels [1]. Figure 5 illustrates a substantial reduction in the expression of *eNOS* mRNA in cells exposed to low shear stress and disturbed flow. In contrast, cells exposed to laminar flow did not show any difference in the mRNA levels when compared to control static cultures. On the other hand, *ET-1* mRNA expression levels dropped significantly by 0.5-fold and 0.8-fold in cells exposed to disturbed and laminar flow, respectively.

### 3.5. ER Stress-Associated Gene Expression

**Figure 6:**
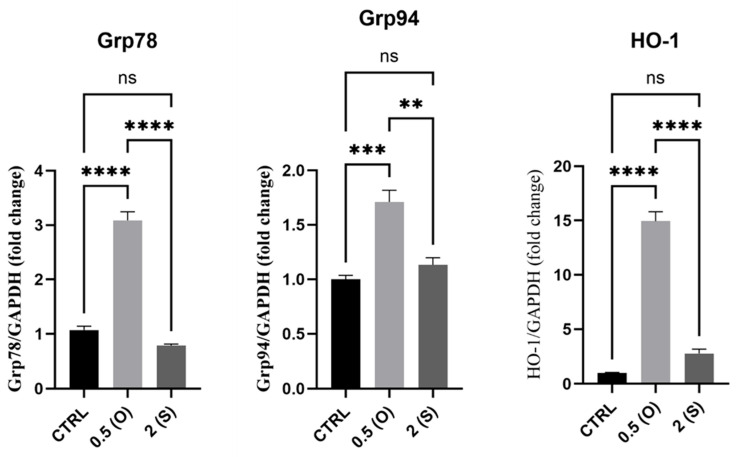
Effects of different flow patterns and shear stresses on mRNA expression of *GRP78*, *GRP94*, and *HO-1*. Relative mRNA expression levels (fold change) of *GRP78*, *GRP94*, and *HO-1* were normalized against the GAPDH housekeeping gene (*n* = 3 in each group). Cells were exposed to two different flow conditions for 24 h. Data are presented as mean ± S.E.M. ** *p* < 0.01, *** *p* < 0.001, **** *p* < 0.0001 versus CTRL or indicated groups. Treatment groups include CTRL, control: static culture with no flow; 0.5(O): oscillatory flow with low shear stress (0.5 dynes/cm^2^); 2(S): laminar flow with high shear stress (2 dynes/cm^2^).

Here, we investigated different genes linked to the ER stress response, including glucose-regulated protein 78 (GRP78), *GRP94*, and heme oxygenase-1 (HO-1). GRP78 mediates several cellular functions, including the transport of freshly synthesized polypeptides across the ER membrane, supporting protein folding, guiding misfolded proteins toward ER-associated degradation (ERAD), controlling calcium homeostasis, and acting as an ER stress sensor. Heat shock protein (HSP) 90 family member GRP94 is a stress-inducible molecular chaperone. The cytoprotective enzyme, HO-1, is frequently induced in response to elevated cellular stress to support the preservation of physiological homeostasis. Many stressors can trigger HO-1 expression. Our results, depicted in Figure 6, demonstrate that mRNA expression of *GRP78*, *GRP94*, and *HO-1* increased in the ECs exposed to disturbed flow and low shear stress. At the same time, there was no significant difference in the expression in laminar flow-exposed cells when compared to the control static cells. It was noteworthy that, for the ECs exposed to laminar flow, the mRNA levels for all the genes studied here were significantly lower compared to those of cells exposed to the disturbed flow pattern.

### 3.6. Effect of Different Flow Patterns and Shear Stresses on ROS Generation

**Figure 7:**
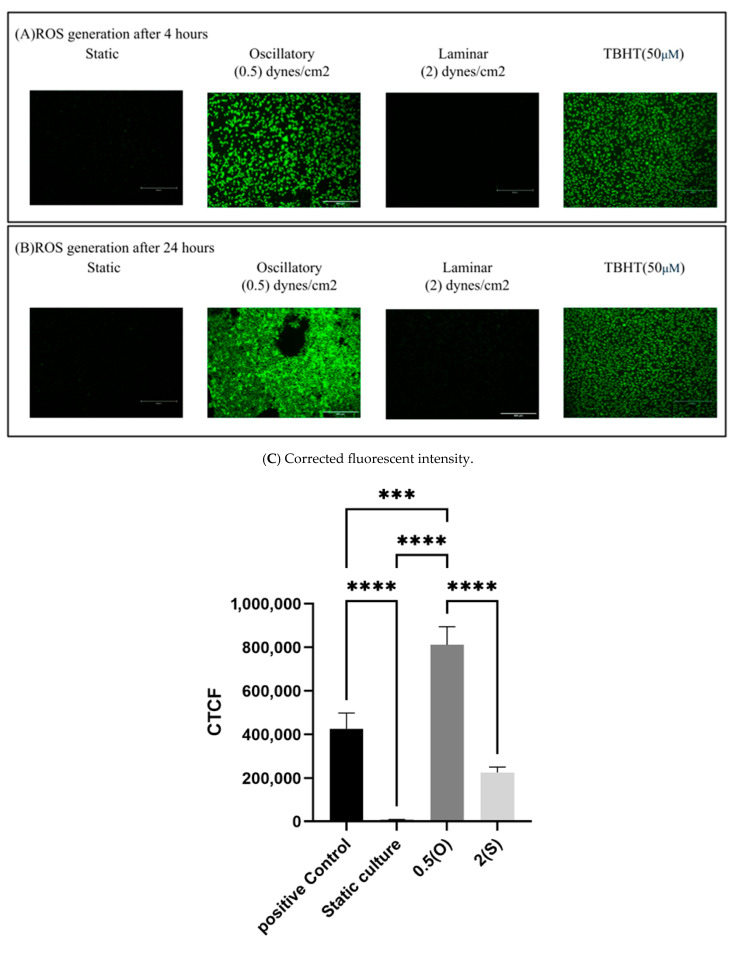

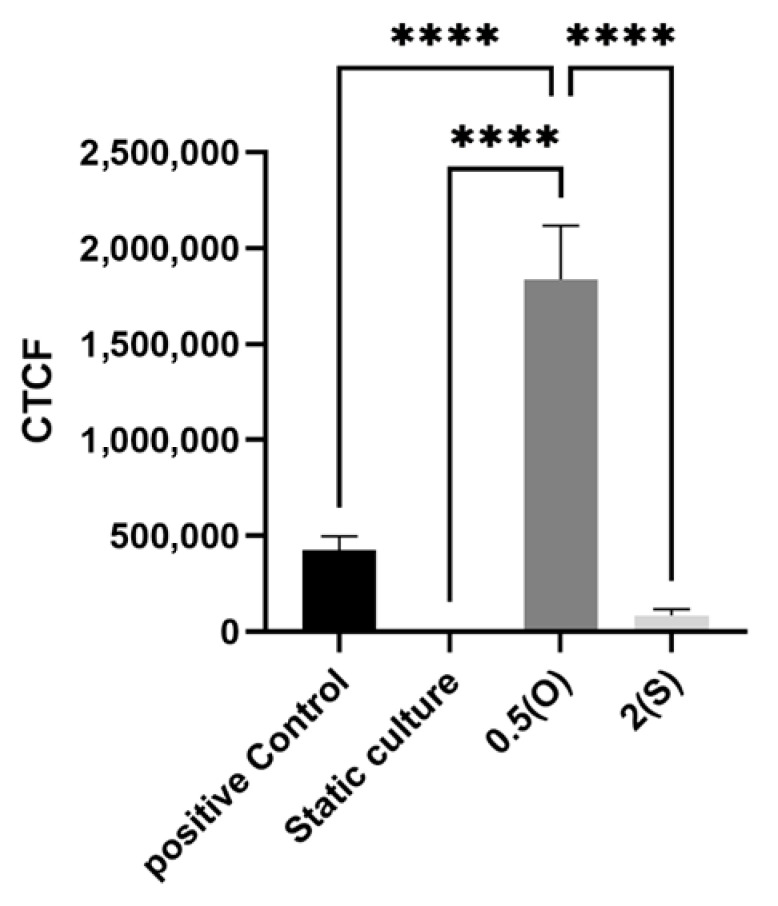
Effect of different flow patterns on levels of intracellular ROS production in EA.hy926 cells. Intracellular ROS production was detected by the DCF fluorescence assay. Representative images of ROS generation observed under a fluorescence microscope at 4 h (**A**) and 24 h (**B**) of exposure to the tested flow patterns; scale bar 600 µm. (**C**) Cells were exposed to two different flow conditions for 4 and 24 h, and the corrected fluorescent intensity was measured. Data are presented as mean ± S.E.M. *** *p* < 0.001, **** *p* < 0.0001 versus CTRL or versus indicated groups. Treatment groups include positive control: TBHT-treated cells, static culture; oscillatory flow with low shear stress (0.5 dynes/cm^2^); and laminar flow with a high shear stress (2 dynes/cm^2^).

Using a 2′,7′-dichlorodihydrofluorescein diacetate DCFDA/H2DCFDA cellular ROS assay kit, the production of ROS was measured in EA.hy926 cells under the tested conditions. Here, a fluorogenic dye called H2DCFDA is used to quantify the activity of ROS (including hydroxyl, peroxyl, and other reactive species) in cells. We exposed the cells to the aforementioned different flow patterns for either 4 h or 24 h. TBHP was used as a positive control in this study. As shown in Figure 7, ECs exposed to disturbed flow with low shear stress demonstrate a significant increase in ROS generated at both 4 and 24 h time-points when compared to statically cultured cells. Laminar flow-treated cells and statically cultured cells did not show ROS generation.

## 4. Discussion

In the current study, we designed and optimized an in vitro model system that can be efficiently used in studies investigating the effect of shear stress as a contributing factor for the development of cardiovascular disease on triggering oxidative stress and endothelial dysfunction, which are the hallmarks of atherosclerosis and aortic aneurysms [1,15]. Here, we began to expose EA.hy926 endothelial cells to various flow patterns (disturbed oscillatory or steady) and shear stresses (0.5 or 2 dynes/cm^2^). In this work, we combined two different flow patterns and magnitudes of shear stresses and assessed signaling pathways regarding anti-oxidant, inflammatory, and ER stress responses. Previous studies have employed various models, such as cone-and-plate chambers, parallel plate chambers, and displacement pumps, to generate different shear stresses or flow patterns [10]. Simple peristaltic pumps, used as a model to investigate the impact of shear stress and flow patterns on the biology of endothelial cells, are considered limited due to the complex flows they generate. Some other studies have only investigated and validated the impact of different patterns of flow using modified or commercial dampeners to generate steady shear stress flows [7]. In our model, we integrated a simple peristaltic pump into a multichannel cell culture chamber to apply low oscillatory shear stress, mimicking the case in atheroprone areas of the vasculature, and relatively high steady shear stress to represent a healthy flow profile. We then assessed the cellular responses and validated the system’s usefulness by analyzing shear stress-induced ER and oxidative stress markers. A peristaltic pump was used to generate two different flow patterns and shear stresses: a steady flow of 2 dynes/cm^2^ was implemented using the syringe dampeners, and a disturbed oscillatory flow of 0.5 dynes/cm^2^ was implemented using the peristaltic pump setup without dampeners since low shear stress is known to trigger ROS generation [1]. Several cellular responses associated with endothelial injury under different flow patterns were investigated. Each studied pathway is discussed in subsequent sections below.

### 4.1. Markers of Cellular Inflammation: NF-κB and COX-2

As our data demonstrated, there was a significant increase in the gene expression of *NF-κB* and *COX-2* in ECs under low shear stress and oscillatory flow. These data align with a study by Huang et al., in which the CX3CR1/NF-κB signaling pathway was activated in vitro in response to low shear stress, eliciting an inflammatory response [16]. In addition, the expression of COX-2 was found to be triggered by an inflammatory stimulus, where endothelial COX-2 expression was upregulated in response to fluid shear stress [17]. It is known that a disturbed flow profile triggers cellular responses that elicit oxidative stress and mitochondrial dysfunction, promoting the proinflammatory response, including Mitogen-Activated Protein Kinase (MAPK) and Toll-Like Receptor (TLR) pathways, which in turn leads to the *COX-2* and *NF-κB* amplified gene expression [18]. In the current study, mRNA expression of *COX-2* was upregulated in ECs under disturbed flow. In contrast, minimal expression was observed in static cultures. Our findings are consistent with preceding research, indicating that different levels of shear stress increase the expression of *COX-2*. It is worth noting that cells subjected to disturbed flow exhibit considerably elevated levels of *COX-2* expression. The upregulation of COX-2 synthesis is believed to have cardioprotective effects in response to stress, particularly in atheroprone areas of blood vessels [19]. ECs utilize mechanosensing mechanisms to translate the flow shear stress they experience into alterations in gene expression. The activation of the transcription factor NF-κB is a well-established consequence of flow [14,20,21]. The NF-κB pathway plays a pivotal role in inflammatory reactions. The activation of proinflammatory machinery significantly elicits atherosclerosis, particularly in areas where low-shear stress and disturbed flow predominate, such as arterial branching sites [14,21].

### 4.2. TGF-β

Arterial remodeling is known to be significantly mediated by fluid shear stress. Low and oscillatory wall shear stress triggers a proinflammatory response in ECs in areas of disturbed flow. It governs atherosclerotic remodeling of the impacted arteries by promoting lipid accumulation and elastin degradation. In a study conducted by Kim et al., a relationship was demonstrated between disturbed flow and TGF-β-mediated stiffening of arteries via the action of the shear-sensitive protein thrombospondin-1 (TSP-1), which is overexpressed under disturbed flow conditions [22]. Compared to static conditions, our results demonstrate a significant reduction in mRNA levels in ECs exposed to both steady and disturbed flow patterns. These findings are consistent with the investigation conducted by Kim et al., which demonstrated that TGF-β1 is reduced in the endothelium but not in the arterial media and adventitia after 24 h of disrupted flow in vivo. In addition, the study also found that human aortic endothelial cells showed decreased mRNA levels of *TGF-β* when subjected to laminar shear, which imitates a constant flow, as opposed to being in a static culture environment [22].

### 4.3. Cell Death Markers: CHOP, C-Jun

Previous studies have provided evidence confirming that disturbed flow and low shear stress increase the mRNA levels of C-Jun [23,24]. Our results are in agreement with the literature, as disturbed flow with low shear stress conditions has been shown to lead to a significant increase in the *C-Jun* mRNA expression in ECs [24,25]. Conversely, we found that ECs under continuous flow exhibited a less substantial rise in mRNA levels compared to static cultures. Contrary to previous data on steady flow patterns, our data demonstrated an increase in mRNA expression compared to static cultures [26]. This difference could be attributed to post-transcriptional effects or the comparatively lower shear stress value in physiological settings. Genes related to ER stress and the unfolded protein response (UPR) are upregulated in ECs of atheroprone regions with disturbed flow and oscillatory shear stress patterns [1]. It is revealed that disturbed flow causes ER stress by activating X-binding protein (XBP)-1 and CHOP, leading ECs to undergo apoptosis [27,28]. This results in a threefold rise in the relative expression of CHOP under disturbed flow compared to laminar flow. The reason for this is that artery cells have a reduced ability to properly fold proteins when exposed to modest wall shear stress (<2 dynes/cm^2^) caused by disrupted flow. Our results align with these findings and corroborating data.

### 4.4. eNOS and ET-1

NO derived from the endothelium is a physiologically important vasodilator that also inhibits adhesion and platelet aggregation. Furthermore, by downregulating leukocyte adhesion to the endothelium, vascular NO can inhibit leukocyte adhesion [29]. NO controls vascular tone, dilatation, and contains the vascular inflammation via blocking NF-κB, which affects Intercellular Adhesion Molecule 1 (ICAM-1) expression. Also, the EC glycocalyx’s sialic acid glycoproteins, heparan sulfate, and hyaluronic acid are all fragmented by disturbed flow. The abnormalities in the expression pattern of these components lead to decreased eNOS expression and impairment of the glycocalyx’s capacity as a mechano-transducer [30]. Additionally, ROS generation by disturbed flow positively affects the endothelium. Bone morphogenetic protein 4 (BMP4) mediates this process by inducing increased superoxide production through NADPH oxidase and eNOS uncoupling-dependent mechanisms [31]. In line with these findings, our data show that ECs exposed to disturbed flow with low shear stress exhibit a significant decrease in *eNOS* mRNA levels compared to static cultures and cells exposed to laminar flow. As expected, the mRNA expression of *eNOS* was not affected in ECs exposed to laminar flow. Our results confirmed the findings documented in the literature, which includes research conducted by Harding et al. This study revealed that *eNOS* expression was twofold higher under normal physiological laminar flow conditions than under disturbed flow conditions. Additionally, the glycocalyx components were disrupted under conditions of disturbed flow, leading to a subsequent decrease in NO generation [32].

ECs primarily secrete endothelin-1 (ET-1) which is a robust endogenous vasoconstrictor. Our results showed a significant reduction in the *ET-1* mRNA levels in both dynamic conditions. A study on the BAECS cell line demonstrated that ECs subjected to unidirectional shear stress temporarily increased the expression of the *ET-1* gene, after a 24-h exposure to the flow; however, this upregulation attenuates entirely. In comparison to static conditions, BAECs tested under oscillatory flow (0.3 ± 3 dynes/cm^2^) demonstrated a significant increase in *ET-1* mRNA expression [33]. Our findings indicate that ECs subjected to a steady flow exhibited behavior consistent with what has been reported in the literature. Nevertheless, ECs subjected to disturbed flow conditions revealed conflicting outcomes. This phenomenon can be related to amplified stress versions, which consequently actively promote programmed cell death and impair ET-1 generation machinery.

### 4.5. ER Stress Markers: GRP94,GRP78, and HO-1

The ER of EC harbors molecular chaperones, including GRP94 and GRP78, which are crucial for ensuring the proper folding of newly synthesized proteins [1]. During the ER stress response, it has been established that disturbed flow directly causes sustained GRP78, spliced XBP-1, and CHOP [31]. Multiple studies have shown that inhibiting the ER stress response, resulting from disrupted blood flow, effectively prevents and rescues atherosclerosis. Nevertheless, there is insufficient data to establish direct connections between laminar flow and ER stress in ECs. Laminar flow was found to hinder the activation of the UPR pathway [32]. The results of our current study are consistent with previous research, which suggests that ECs that are subjected to low shear stress and disturbed flow show significantly increased levels of GRP78 and GRP94. The rise in levels is caused by oxidative stress and the triggering of the UPR in settings that imitate environments prone to atherosclerosis. In contrast, ECs exposed to a continuous/steady flow did not show any notable variation compared to control cultures maintained in a stationary state.

In ECs, HO-1 has substantial anti-inflammatory, anti-apoptotic, antiproliferative, immunomodulatory, and antioxidant properties, the majority of which are crucial for the athero-protective function of HO-1 [33]. It is well documented that laminar shear stress provides additional evidence for the protective function of HO-1, as laminar shear stress can activate antioxidant genes, including HO-1. In contrast, ECs exposed to disturbed shear demonstrate a diminished level of HO-1 [34,35]. The findings of our study indicate a considerable upregulation of HO-1 mRNA levels in cells subjected to low shear stress and disturbed flow, as compared to static cultures and ECs exposed to steady flow. The observed rise in levels can be attributed to the pro-oxidant conditions that are caused by the disruption of flow and the decrease in shear stress. Oxidative stress and ischemic conditions are broadly documented as activators of HO-1, which possesses anti-apoptotic and cell-protective properties [36]. However, the elevation of HO-1 mRNA levels does not automatically produce functional proteins that have antioxidant or anti-inflammatory effects, possibly due to their inherent instability. According to the data mentioned earlier, the flow patterns designated efficiently replicated the events that promote atherosclerosis progression via inducing cellular oxidative stress.

### 4.6. ROS Generation

The multi-step process of atherosclerosis involves extended periods of inflammation and elevated ROS generation. Serving as second messengers, ROS may activate several downstream signaling pathways that ultimately result in atherosclerosis. ROS levels are enhanced by low-shear stress. In addition to the gene expression findings, we examined the oxidative stress levels in ECs exposed to various flow patterns and magnitudes of shear stress using a fluorescent assay. The findings of our study indicate a significant increase in the production of ROS in ECs when subjected to disrupted flow and mild shear stress, for both 4- and 24-h durations. However, ECs subjected to a steady flow for 24 h showed a negligible rise in ROS levels as compared to static cultures. Under conditions of steady flow, evidence suggests that it enhances an environment that prevents the development of atherosclerosis by increasing the expression of genes that produce antioxidants. Consequently, a decrease in levels of ROS and pro-oxidant activity results in a state that is overall more resistant to oxidative damage.

In contrast, disturbed flow patterns result in elevated levels of ROS and enhanced pro-oxidant activity, while also causing a decrease in the availability of NO and a reduction in antioxidant activity. This induces an oxidative state that supports the stimulation and control of important transcription factors, such as NF-κB, ultimately encouraging a pro-atherogenic milieu by enhancing the production of Monocyte Chemoattractant Protein-1(MCP-1) [34,35]. This work aimed to verify the effectiveness of a cell culture hemodynamic system in replicating atheroprone settings. We achieved this by evaluating the impact of low shear stress and disrupted flow patterns, as well as steady flow with increased shear stress, over a 24-h period. This model enables the study of flow-associated disorders, such as atherosclerosis and aortic aneurysms, by utilizing a straightforward peristaltic pump, precise shear stress values, and predetermined exposure times to provide the required hemodynamic circumstances. The results of our study provide vital information on genes involved in sensing mechanical forces and the state of oxidative stress. In situations of laminar flow, the impact on oxidative stress and gene expression is triggered by the laminar flow pattern rather than the actual magnitude of shear stress. Compared to static cultures, laminar flow led to a negligible increase in ROS generation and several evaluated gene levels.

### 4.7. Novelty of the Current Study and Contribution to the Existing Knowledge

There are several important points in the current study that contribute to existing knowledge and practice in the literature relevant to in vitro investigations of atherosclerotic diseases. The microfluidic system employed in our study represents a low-cost, custom-designed pump that can generate well-controlled flow and shear stress profiles in EA.hy926 cells, which were cultured on a plastic microfluidic chip without the need for coating with ECM-mimicking materials. These cells represent a well-established model in studies dissecting endothelial cell behavior and responses to pathological and physiological flow conditions. Using EA.hy926 cells as immortalized cells is advantageous as an in vitro model due to their ability for unlimited proliferation and cost-effectiveness. They are free from variability associated with primary endothelial cell lines, making them ideal for high-throughput studies [36,37]. In our study, to ensure consistency, we used EA.hy926 cells up to passage 5. Our developed system confirmed, with quantitative evidence, that distinct hemodynamic patterns and shear stress profiles affect antioxidant status, validating the capacity to discriminate between atheroprone and atheroprotective flow environments. Moreover, our simple model offers high reproducibility compared to commercial systems, which usually lack accessibility or adaptability. Significantly, exposing EA.hy926 cells to low shear stress and disturbed flow using our setup consistently elicits signatures of ER stress, inflammatory markers, and ROS generation; on the other hand, exposing cells to relatively low shear stress with laminar steady flow pattern exhibited a normal oxidative status, normal eNOS gene expression, unchanged expression of ER stress markers, and absence of elevated ROS generation detected; this confirms the results of a previous study [7]. We interpret this as a flow profile that simulates the chronic shear stress, which is key in inducing cytoprotective phenotypes in endothelial cells. This cytoprotective profile can also explain the slight increase in Cox-2, an inflammatory marker that plays a protective role against apoptosis in some cases [38], thereby protecting the cell from dysfunction. Two distinct shear stress levels and two distinct flow profiles were applied: 0.5 dynes/cm^2^ oscillatory flow and 2 dynes/cm^2^ steady laminar flow. While atherosclerosis predominantly occurs in large arteries, such as the aorta, it may also affect medium to small arteries, including the coronary arteries, which are a few millimeters in diameter. Shear stress values investigated in our study are considered low, as previous studies define them to be less than 10 dynes/cm^2^ in coronary arteries under certain pathological conditions [39,40]. While the magnitude difference between these two cases is not too drastic (i.e., laminar shear is about 4 times the oscillatory shear), this difference was sufficient to induce significantly different gene expression patterns, emphasizing the importance of hemodynamics within small arteries in both healthy and diseased conditions.

## 5. Conclusions

The current work aims to design a refined system for investigating the influence of shear stress on hemodynamics, cellular oxidative stress, and endothelial dysfunction, which are crucial elements in the progression of atherosclerosis and aortic aneurysms. Our cell culture hemodynamic system, which employs a peristaltic pump, accurately replicates atheroprone circumstances. By subjecting ECs to oscillatory and laminar flows with different shear stress values, we demonstrated significant changes in gene expression associated with inflammation, endothelial function, and oxidative stress. This optimized model offers a valuable tool for investigating the molecular pathways underlying flow-related disorders.

## Figures and Tables

**Table 1:** List of human primer pair sequences.

Target	Forward	Reverse
*GRP78*	CATCACGCCGTCCTATGTCG	CGTCAAAGACCGTGTTCTCG
*GRP94*	GCTGACGATGAAGTTGATGTGG	CATCCGTCCTTGATCCTTCTCTA
*CHOP*	GAACGGCTCAAGCAGGAAATC	TTCACCATTCGGTCAATCAGAG
*HO-1*	AAGACTGCGTTCCTGCTCAAC	AAAGCCCTACAGCAACTGTCG
*c-Jun*	CCTTGAAAGCTCAGAACTCGGAG	TGCTGCGTTAGCATGAGTTGGC
*NFκ-B*	GCAGCACTACTTCTTGACCACC	TCTGCTCCTGAGCATTGACGTC
*COX-2*	CGGTGAAACTCTGGCTAGACAG	GCAAACCGTAGATGCTCAGGGA
*GAPDH*	CCAAGGAGTAAGACCCCTGG	TGGTTGAGCACAGGGTACTT
*TGFβ*	CCCAGCATCTGCAAAGCTC	GTCAATGTACAGCTGCCGCA
*eNOS*	GAAGGCGACAATCCTGTATGGC	TGTTCGAGGGACACCACGTCAT
*ET-1*	CTACTTCTGCCACCTGGACATC	TCACGGTCTGTTGCCTTTGTGG

## Data Availability

The original contributions presented in this study are included in the article. Further inquiries can be directed to the corresponding author.

## References

[1] Hasan M., Al-Thani H., El-Menyar A., Zeidan A., Al-Thani A., Yalcin H.C. (2023). Disturbed hemodynamics and oxidative stress interaction in endothelial dysfunction and AAA progression: Focus on Nrf2 pathway. Int. J. Cardiol..

[2] Katoh K. (2023). Effects of Mechanical Stress on Endothelial Cells In Situ and In Vitro. Int. J. Mol. Sci..

[3] Mutlu O., Salman H.E., Al-Thani H., El-Menyar A., Qidwai U.A., Yalcin H.C. (2023). How does hemodynamics affect rupture tissue mechanics in abdominal aortic aneurysm: Focus on wall shear stress derived parameters, time-averaged wall shear stress, oscillatory shear index, endothelial cell activation potential, and relative residence time. Comput. Biol. Med..

[4] Salman H.E., Yalcin H.C. (2021). Computational Modeling of Blood Flow Hemodynamics for Biomechanical Investigation of Cardiac Development and Disease. J. Cardiovasc. Dev. Dis..

[5] Cavallero S., Blázquez-Medela A.M., Satta S., Hsiai T.K. (2021). Endothelial mechanotransduction in cardiovascular development and regeneration: Emerging approaches and animal models. Curr. Top. Membr..

[6] Mazhar N., Islam M.S., Raza M.Z., Mahin S.K.H., Islam M.R., Chowdhury M.E.H., Al-Ali A., Agouni A., Yalcin H.C. (2024). Comparative Analysis of In Vitro Pumps Used in Cardiovascular Investigations: Focus on Flow Generation Principles and Characteristics of Generated Flows. Bioengineering.

[7] Warabi E., Wada Y., Kajiwara H., Kobayashi M., Koshiba N., Hisada T., Shibata M., Ando J., Tsuchiya M., Kodama T. (2004). Effect on endothelial cell gene expression of shear stress, oxygen concentration, and low-density lipoprotein as studied by a novel flow cell culture system. Free Radic. Biol. Med..

[8] Chen X.L., Varner S.E., Rao A.S., Grey J.Y., Thomas S., Cook C.K., Wasserman M.A., Medford R.M., Jaiswal A.K., Kunsch C. (2003). Laminar flow induction of antioxidant response element-mediated genes in endothelial cells. A novel anti-inflammatory mechanism. J. Biol. Chem..

[9] Goettsch C., Goettsch W., Brux M., Haschke C., Brunssen C., Muller G., Bornstein S.R., Duerrschmidt N., Wagner A.H., Morawietz H. (2011). Arterial flow reduces oxidative stress via an antioxidant response element and Oct-1 binding site within the NADPH oxidase 4 promoter in endothelial cells. Basic Res. Cardiol..

[10] Pech S., Richter R., Lienig J. Peristaltic Pump with Continuous Flow and Programmable Flow Pulsation. Proceedings of the 2020 IEEE 8th Electronics System-Integration Technology Conference (ESTC).

[11] McSweeney S.R., Warabi E., Siow R.C.M. (2016). Nrf2 as an Endothelial Mechanosensitive Transcription Factor. Hypertension.

[12] Booth R., Noh S., Kim H. (2014). A multiple-channel, multiple-assay platform for characterization of full-range shear stress effects on vascular endothelial cells. Lab Chip.

[13] Orr A.W., Hahn C., Blackman B.R., Schwartz M.A. (2008). p21-Activated Kinase Signaling Regulates Oxidant-Dependent NF-κB Activation by Flow. Circ. Res..

[14] Yan H., Hu Y., Akk A., Wickline S.A., Pan H., Pham C.T.N. (2022). Peptide-siRNA nanoparticles targeting NF-κB p50 mitigate experimental abdominal aortic aneurysm progression and rupture. Biomater. Adv..

[15] Fatima M.T., Hasan M., Abdelsalam S.S., Sivaraman S.K., El-Gamal H., Zahid M.A., Elrayess M.A., Korashy H.M., Zeidan A., Parray A.S. (2021). Sestrin2 suppression aggravates oxidative stress and apoptosis in endothelial cells subjected to pharmacologically induced endoplasmic reticulum stress. Eur. J. Pharmacol..

[16] Huang H., Ren P., Zhao Y., Weng H., Jia C., Yu F., Nie Y. (2023). Low shear stress induces inflammatory response via CX3CR1/NF-κB signal pathway in human umbilical vein endothelial cells. Tissue Cell.

[17] Russell-Puleri S., Dela Paz N.G., Adams D., Chattopadhyay M., Cancel L., Ebong E., Orr A.W., Frangos J.A., Tarbell J.M. (2017). Fluid shear stress induces upregulation of COX-2 and PGI(2) release in endothelial cells via a pathway involving PECAM-1, PI3K, FAK, and p38. Am. J. Physiol. Heart Circ. Physiol..

[18] Pizzino G., Irrera N., Cucinotta M., Pallio G., Mannino F., Arcoraci V., Squadrito F., Altavilla D., Bitto A. (2017). Oxidative Stress: Harms and Benefits for Human Health. Oxid. Med. Cell Longev..

[19] Funk C.D., FitzGerald G.A. (2007). COX-2 Inhibitors and Cardiovascular Risk. J. Cardiovasc. Pharmacol..

[20] Fang Y., Wu D., Birukov K.G. (2019). Mechanosensing and Mechanoregulation of Endothelial Cell Functions. Compr. Physiol..

[21] Guo Q., Jin Y., Chen X., Ye X., Shen X., Lin M., Zeng C., Zhou T., Zhang J. (2024). NF-κB in biology and targeted therapy: New insights and translational implications. Signal Transduct. Target. Ther..

[22] Kim C.W., Pokutta-Paskaleva A., Kumar S., Timmins L.H., Morris A.D., Kang D.-W., Dalal S., Chadid T., Kuo K.M., Raykin J. (2017). Disturbed Flow Promotes Arterial Stiffening Through Thrombospondin-1. Circulation.

[23] Cuhlmann S., Heiden K.V.d., Saliba D., Tremoleda J.L., Khalil M., Zakkar M., Chaudhury H., Luong L.A., Mason J.C., Udalova I. (2011). Disturbed Blood Flow Induces RelA Expression via c-Jun N-Terminal Kinase 1. Circ. Res..

[24] Lee J.Y., Chung J., Kim K.H., An S.H., Kim M., Park J., Kwon K. (2018). Fluid shear stress regulates the expression of Lectin-like oxidized low density lipoprotein receptor-1 via KLF2-AP-1 pathway depending on its intensity and pattern in endothelial cells. Atherosclerosis.

[25] Wang J., An F.S., Zhang W., Gong L., Wei S.J., Qin W.D., Wang X.P., Zhao Y.X., Zhang Y., Zhang C. (2011). Inhibition of c-Jun N-terminal kinase attenuates low shear stress-induced atherogenesis in apolipoprotein E-deficient mice. Mol. Med..

[26] Liu Y., Yin G., Surapisitchat J., Berk B.C., Min W. (2001). Laminar flow inhibits TNF-induced ASK1 activation by preventing dissociation of ASK1 from its inhibitor 14-3-3. J. Clin. Invest..

[27] Chung J., Kim K.H., Lee S.C., An S.H., Kwon K. (2015). Ursodeoxycholic Acid (UDCA) Exerts Anti-Atherogenic Effects by Inhibiting Endoplasmic Reticulum (ER) Stress Induced by Disturbed Flow. Mol. Cells.

[28] Zeng L., Zampetaki A., Margariti A., Pepe A.E., Alam S., Martin D., Xiao Q., Wang W., Jin Z.-G., Cockerill G. (2009). Sustained activation of XBP1 splicing leads to endothelial apoptosis and atherosclerosis development in response to disturbed flow. Proc. Natl. Acad. Sci. USA.

[29] Kleinert H., Forstermann U., Enna S.J., Bylund D.B. (2007). Endothelial Nitric Oxide Synthase. xPharm: The Comprehensive Pharmacology Reference.

[30] Sunderland K., Jiang J., Zhao F. (2022). Disturbed flow’s impact on cellular changes indicative of vascular aneurysm initiation, expansion, and rupture: A pathological and methodological review. J. Cell Physiol..

[31] Heo K.S., Fujiwara K., Abe J. (2011). Disturbed-flow-mediated vascular reactive oxygen species induce endothelial dysfunction. Circ. J..

[32] Harding I.C., Mitra R., Mensah S.A., Herman I.M., Ebong E.E. (2018). Pro-atherosclerotic disturbed flow disrupts caveolin-1 expression, localization, and function via glycocalyx degradation. J. Transl. Med..

[33] da Silva R.F., Chambaz C., Stergiopulos N., Hayoz D., Silacci P. (2007). Transcriptional and post-transcriptional regulation of preproendothelin-1 by plaque-prone hemodynamics. Atherosclerosis.

[34] Chatterjee S., Browning E.A., Hong N., DeBolt K., Sorokina E.M., Liu W., Birnbaum M.J., Fisher A.B. (2012). Membrane depolarization is the trigger for PI3K/Akt activation and leads to the generation of ROS. Am. J. Physiol. Heart Circ. Physiol..

[35] Hsieh H.J., Liu C.A., Huang B., Tseng A.H., Wang D.L. (2014). Shear-induced endothelial mechanotransduction: The interplay between reactive oxygen species (ROS) and nitric oxide (NO) and the pathophysiological implications. J. Biomed. Sci..

[36] Ruze A., Zhao Y., Li H., Gulireba X., Li J., Lei D., Dai H., Wu J., Zhao X., Nie Y. (2018). Low shear stress upregulates the expression of fractalkine through the activation of mitogen-activated protein kinases in endothelial cells. Blood Coagul. Fibrinolysis.

[37] Balaguru U.M., Sundaresan L., Manivannan J., Majunathan R., Mani K., Swaminathan A., Venkatesan S., Kasiviswanathan D., Chatterjee S. (2016). Disturbed flow mediated modulation of shear forces on endothelial plane: A proposed model for studying endothelium around atherosclerotic plaques. Sci. Rep..

[38] Shi Z., Chen Y., Pei Y., Long Y., Liu C., Cao J., Chen P. (2017). The role of cyclooxygenase-2 in the protection against apoptosis in vascular endothelial cells induced by cigarette smoking. J. Thorac. Dis..

[39] Samady H., Eshtehardi P., McDaniel M.C., Suo J., Dhawan S.S., Maynard C., Timmins L.H., Quyyumi A.A., Giddens D.P. (2011). Coronary Artery Wall Shear Stress Is Associated With Progression and Transformation of Atherosclerotic Plaque and Arterial Remodeling in Patients With Coronary Artery Disease. Circulation.

[40] Aboyans V., Lacroix P., Criqui M.H. (2007). Large and Small Vessels Atherosclerosis: Similarities and Differences. Prog. Cardiovasc. Dis..

